# Surgical site infections after cesarean sections at the University Clinical Center of Kosovo: rates, microbiological profile and risk factors

**DOI:** 10.1186/s12879-019-4383-7

**Published:** 2019-08-28

**Authors:** Vjosa A. Zejnullahu, Rozalinda Isjanovska, Zana Sejfija, Valon A. Zejnullahu

**Affiliations:** 1 Department of Obstetrics and Gynecology, University Clinical Center of Kosovo, Pristina, Kosovo; 2 Institute of Epidemiology, Biostatistics and Medical Informatics, Ss. Cyril and Methodius University, Skopje, Macedonia; 3 Department of Oral Surgery, University Clinical Center of Kosovo, Pristina, Kosovo; 4 Departments of Abdominal Surgery, University Clinical Center of Kosovo, Pristina, Kosovo

**Keywords:** Surgical site infections, Cesarean delivery, Antibiotics, Bacteriological profile, Kosovo

## Abstract

**Background:**

Surgical site infections (SSI) are a common complication after a cesarean section (C-section) and mainly responsible for increased maternal mortality and morbidity, dissatisfaction of patients, longer hospital stays as well as higher treatment costs. The aim of this study is to determine the incidence rate and risk factors of surgical site infections in women undergoing caesarean section at the University Clinical Center of Kosovo (UCCK), in the Clinic for Obstetrics and Gynecology.

**Methods:**

We conducted a prospective observational cohort study involving 325 women who underwent labor and scheduled C-sections from January, 2018 to September, 2018 at the University Clinical Center of Kosovo, Clinic for Obstetrics and Gynecology. Each woman was followed for 30-postoperative days. Data analysis included descriptive statistics, univariate and multivariate logistic regression analysis. Culture-based microbiological methods were used to identify causal agents in postoperative wounds.

**Results:**

Overall the SSI rate was 9.85% and the median time to SSI was the 7th postoperative day. The mean age of the patients was 31.3 ± 5.5 years (range from 17 to 46 years). The average length of stay was 4.2 ± 3.4 days. Several factors reduced the risk of SSI. These included: age less than 35 years (RR 0.25; 95% CI; 0.199–0.906 and *P* = 0.027) preoperative use of antibiotics (RR 0.232; 95% CI; 0.107–0.502 and *P* = 0.000) and duration of the operation less than 1 h (RR 0.135; 95% CI; 0.054–0.338 and *P* = 0.000). Previous cesarean section and one or more co-morbidity were associated with 7.4 fold and 8 fold increased risk of SSI, respectively. We found a statistically significant association between SSI and co-morbidity, preoperative antibiotic use, duration of operation, age and history of previous cesarean section (*P* = 0.000; 0.000; 0.0001; 0.023; 0.000; respectively using chi-square test). Multivariable logistic regression analysis confirmed that one or more co-morbidity, previous C-section, preoperative antibiotics and duration of the surgery < 1 h are predictors of SSI.

**Conclusion:**

The high incidence rate of SSIs after C-sections in this study highlight the need for prioritizing SSI control and surveillance. Patient demographics, procedures utilized and surgical factors must be incorporated in programs to reduce the infection rate. Additionally, an effort must be given to decrease number of the C-sections performed for the first time through assuring optimal care for the mother and child. The National Committee for Prevention and Control of Nosocomial infection in Kosovo should provide updated guidelines for control and prevention of the nosocomial infections.

## Background

Health care-associated infections (HAIs) or previously named nosocomial infections include: central line-associated bloodstream infections, catheter–associated urinary tract infections, surgical site infections and ventilator-associated pneumonia [1]. Results from recent point prevalence research reported surgical site infections (SSIs) as the most prevalent health-care associated infection [2].

The Surgical Site Infection (SSI) is defined by the Disease Control and Prevention (CDC) criteria as an infection which occurs within 30 days after a surgical procedure and is further divided into superficial incisional primary and secondary SSIs, deep incisional primary and secondary SSIs and organ/space SSIs if involving structures deeper than muscle and fascia space [3].

SSIs are associated with increased costs, higher rates of patient dissatisfaction, increased length of hospital stay and high mortality and morbidity rates [4]. It is estimated that by using an evidence-based approached half of the surgical site infections can be prevented [4].

The National Healthcare Safety Network (NHSN) reported an overall SSIs rate of 1.9% from 2006 to 2008 with 16,147 SSIs in 849,659 operations [5]. Globally rates of SSIs differ substantially and are higher in less developed countries, compared to more developed countries where advanced hospital infection control services exist and correct implementation of evidence–based guidelines for SSI prevention are functional [6]. The World Health Organization (WHO) recommends C-section rates between 10 and 15% since 1985 [7]. There is an increased rate of C-sections in Kosovo following global trends, and therefore surgical site infections after cesarean delivery presents an essential issue to address. In Kosovo, the rate of C-sections rose from 7.5% in 2000 to 27.3% in 2015. In the tertiary referral care service in Kosovo, 33.5% of deliveries are cesarean sections. The highest percentage is noted in private hospitals where the C-section rate is 58% based on data from the annual report from the National Institute of Public Health of Kosovo, 2018 and Ministry of Health of Kosovo.

The Robson classification system to monitor and compare C-section rates within and between services is not used in Kosovo [7].

Preexisting morbidities associated with SSIs include: obesity, smoking, blood transfusion, age, malnutrition, immune incompetence, immunosuppressive therapy, longer preoperative hospitalization, and diabetes mellitus [8]. Factors specifically related to C-sections include: lack of prenatal care, multiple pregnancies, history of previous C- section, chorioamnionitis, pre-labor rupture of the fetal membranes, labor dystocia, emergency/labored delivery, and obstetrical service performed in the teaching hospitals [9].

The Obstetrics/Gynecology Clinic of University Clinical Center of Kosovo is the only tertiary referral care service in Kosovo with 13 Departments and a capacity of 422 beds. The clinic has an average number of 10,000 deliveries per year, as well as an average of 4729 obstetric and gynecologic surgeries performed each year. As in other less developed countries, in Kosovo one of the major obstacles in the control and prevention of the SSIs and other Healthcare Associated Infections (HAIs), is the absence of well-established programs for infection surveillance. In a prospective cohort study conducted by Raka and colleagues, the SSI rate in the Department of abdominal surgery within the University Clinical Center of Kosovo was 12% [10]. The National Committee for Prevention and Control of Nosocomial Infection was established in Kosovo in 2006. Yet active monitoring and effective periodic surveillance in regional hospitals and Clinics within the University Clinical Center, with guidelines to increase the index of the health care system by decreasing the rate of nosocomial infections, remains a future challenge [10].

In their previous study, Raka et al. there were only 11 cases from Gynecology/Obstetric Ward at UCCK and 3 cases of deep surgical site infections caused by *Enterococcus species*. The overall prevalence rate of nosocomial infections in high risk units in the tertiary care service in Kosovo was 17.4% [11].

However to date, no specific data exist for the incidence rate, risk factors and prevalent microbiological pathogens following obstetrical procedures in the UCCK clinics.

There are often insufficient data in less developed countries to determine the SSI rate and risk factors and often there are no post discharge surveillance programs. The aim of this study is to determine the incidence rate and risk factors of SSIs in the UCCK Obstetric Clinics in women undergoing operative delivery by conducting a post-discharge surveillance program.

## Methods

We conducted a prospective observational cohort study to evaluate the rate, risk factors and microbiological profile at the Clinic for Obstetrics and Gynecology in Pristina, Kosovo (a tertiary referral center).

From January 2018 to September 2018 pregnant women who underwent elective and emergency C-sections were enrolled in the study and followed for 30 days postoperatively.

Data were collected according to the established research protocol. On day of admission, the medical chart was completed for the independent variables: age, place of living, preexisting conditions, diabetes mellitus, anemia, obesity, hypertension as well as admission diagnosis.

After surgery, data documenting pre and postoperative antibiotic prophylaxis, duration of the surgery, type of surgery, ASA score, general endotracheal or regional spinal anesthesia, were prospectively extracted from the medical charts, anesthesia list, patients’ medication list and from the discharge list. Currently, pre-operative antibiotic prophylaxis regimen at the Clinic include the administration of a single 1 g dose Cefazolin to women within a period of 30–60 min prior to the incision followed by repeated 1 g dose cefazolin and a 240 mg dose gentamycin up to the third postoperative day.

It is important to note that the internal hospital policy in our Clinic to provide antibiotic prophylaxis before C-section is not in accordance with the Society of Obstetricians and Gynecologists of Canada (SOGC) clinical practice guidelines for antibiotic prophylaxis in obstetric procedures [12]. SOGC recommends that all women undergoing elective or emergency C-section should receive antibiotic prophylaxis (I-A) and the choice of antibiotic for C-section should be a single dose of a first-generation cephalosporin. Clindamycin or erythromycin can be used if the patient has a penicillin allergy (I-A). In case of lengthy procedure or estimated blood loss > 1.5 l, an additional dose of the antibiotics may be given 3–4 h after the initial dose (III-L) [12].

Clinic policy is to discharge the patient in the third postoperative day after C-section and after the 5th day following the gynecological procedure if the patient is in good postoperative condition. Patients return on the 10th day for reassessment/evaluation. According to the study protocol all subjects participating in the study were followed until 30 days after the operative procedure. Follow-up took place in the Outpatient Department within the Clinic, since there is no other surveillance program for nosocomial infection. Culture-based microbiological methods were used to identify causal agents in postoperative wounds. Material was taken using sterile swabs and then forwarded to the Department of Microbiology. All women who underwent operative delivery were eligible for the study. Excluded from the study were patients who: declined informed consent, were operated in private hospitals and then subsequently hospitalized in our Clinic and those who received antibiotics preoperatively (i.e. premature rupture of membranes).

Surgical site infection (SSI) was determined based on fulfilling one of the following criteria adapted from the Centers for Disease Control and Prevention (CDC) [3].

SSI was diagnosed by a gynecologist during the 30-postoperative days in the Outpatient Department and assigned to the superficial incisional SSI group after meeting at least one of the following criteria as per CDC definition (a) purulent drainage from the superficial incision (b) microorganisms identified by a culture (c) superficial incision deliberately opened by the surgeon or dehisce and culture based testing of the superficial incision or subcutaneous tissue is not performed and if patient had at least one of the following signs or symptoms: localized pain or tenderness; localized swelling; erythema; or heat [3].

Deep incisional SSI involving deep soft tissues of the incision (fascial and muscle layer) was diagnosed by a gynecologist within 30 postoperative days after meeting one of the following criteria purulent drainage from the deep incision, deep incision spontaneously dehisces, microbial identification by culture and an abscess or other evidence of infection involving the deep incision that is detected on gross anatomical exam [3]. Organ /Space SSI was defined as infection involving any part of the body deeper than the fascial/muscle layer or endometritis as specific site of an Organ /Space SSI [3].

### Statistical analysis

Statistical data analysis was performed using SPS Statistics 20 version 10. Descriptive statistics with percentages, mean, standard deviation, frequencies were used for patient, surgical and procedure related factors. Univariate and multivariate logistic regression analysis was used to identify predictors for SSI development. *P*-value less than 0.05% was considered statistically significant.

### Ethics

This research was approved by the Ethical Review Committee, University Clinical Centre of Kosovo with reference number 01/135/19. All research was conducted assuring confidentiality of the research data.

## Results

**Fig. 1:**
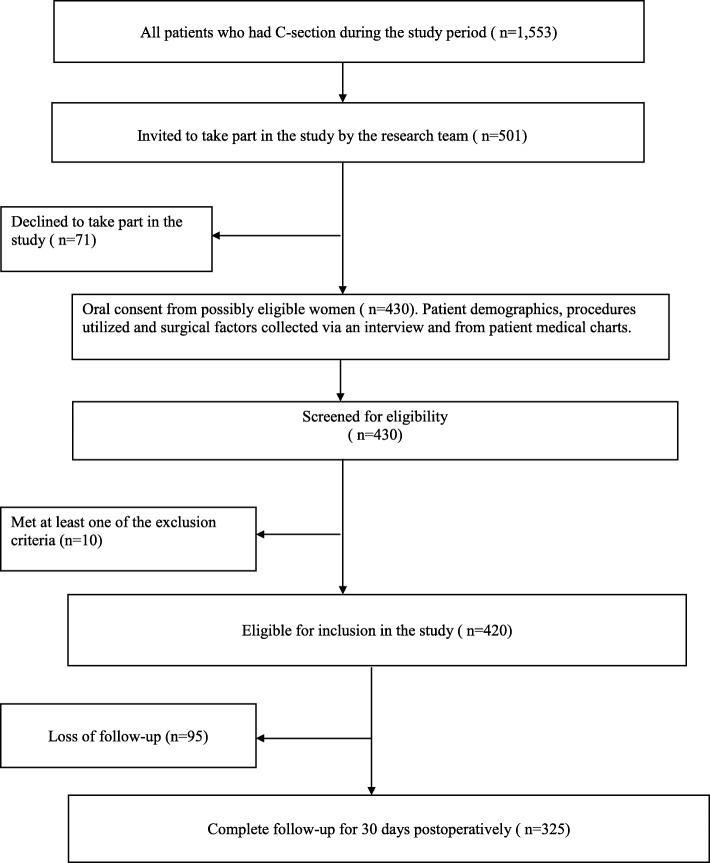
Flowchart of the patient enrollment, follow-up and data collection

A total of 325 cases that met inclusion criteria were followed for 30-postoperative days and analyzed. There were 430 subjects originally recruited into the study. Ten women met initial exclusion criteria and there were 95 lost to follow-up. Reasons for loss of follow-up included: being out of the country at time of follow-up, a decision to withdraw from the study, and pursuing post-operative check-ups at regional hospitals, rather than in Pristina, which were closer to the home of the subjects. Details of the patient enrollment, follow-up and data collection are provided in Fig. 1. The mean age of the patients was 31.3 ± 5.5 years, with a range from 17 to 46 years. More than half, 210 patients (64.6%) were from urban areas and 115 patients (35.4%) were from rural areas. The average length of stay was 4.2 ± 3.4 days. The leading procedure was C-section for the first time in 85.9% of cases, while repeated C-section for the second or more times was found in 14.1% of the study subjects. Of the total number 46 patients (14.1%) have had a history of previous C-section. Three hundred five patients (93.8%) underwent regional anesthesia, the leading anesthesia procedure in the Clinic. Thirty-five patients or 10.8% had one or more co-morbidity namely, hypertensive disease 17 (5.2%), anemia 16 (4.9%), diabetes 6 (1.8%), tuberculosis 1 (0.3%) and five patients (1.5%) were obese. Preoperative antibiotic prophylaxis was identified in 214 patients (65.8%) (Table 1).

**Table 1:** Patient demographics, procedures utilized and surgical factors at the UCC of Kosovo, Clinic for Obstetrics and Gynecology (*N* = 325)

Variables	Mean	Minimum	Maximum	Std.Dev.
Age	31.3	17.0	46.0	5.516561
Living place	No		%	
Urban	210		64.6	
Rural	115		35.4	
Co-morbidity: yes				
Diabetes	6		1.8	
Anemia	16		4.9	
Obesity	5		1.5	
Hypertensive disease/preeclampsia	17		5.2	
Hypothyroidism	1		0.3	
Hypoproteinemia	1		0.3	
Tuberculosis (TBC)	1		0.3	
Previous C-section				
Yes	46		14.1	
Preoperative antibiotics				
Yes	214		65.8	
Postoperative antibiotics				
Yes	325		100.0	
Anaesthesia				
Regional	305		93.8	
General	20		6.2	
Days in hospital – postoperatively^a^	4.2	2.0	29.0	3.394804
SSI/group^b^	11.2		7.8	
without SSI/group^c^	3.4		0.6	
Duration of operation				
> 1 h	134		41.2	
< 1 h	191		58.8	
Emergency (labored) / Elective(scheduled) C-section				
Emergency	102		31.4	
Elective	223		68.6	

^a^ Mann-Whitney U test (U = 1061.5, Z = 70,969,348, *p* = 0.000)

^b^Group with surgical site infection (SSI) ^c^ Group without surgical site infection

Postoperative wound infection was identified in 32 patients comprising 9.85% of the studied subjects (overall SSI rate 9.85%). From this number superficial primary incisional surgical site infection was predominant with 30 cases (93.75%) and deep primary incisional surgical site infection was present in 2 cases (6.25%) while no Organ/Space SSI was identified.

We found statistically significant associations between SSI versus co-morbidity, preoperative antibiotic use, duration of operation, age and history of previous C-section (*P* = 0.000; 0.000; 0.0001; 0.023; 0.000; respectively using chi-square test) (Table 2).

**Table 2:** Univariate logistic regression analysis on predictors for SSI at the UCC of Kosovo, Clinic for Obstetrics and Gynecology

Variables ^a^	B	S.E.	Wald	Df	Sig.	Exp(B)	95% C.I. for EXP(B)	
							Lower	Upper
Age < 35	−0.856	0.386	4.912	1	0.027	0.425	0.199	0.906
Place of living	−0.206	0.4	0.265	1	0.607	0.814	0.371	1.784
Co-morbidity (yes)	2.132	0.423	25.426	1	0.000	8.428	3.681	19.3
Antibiotics (yes)	−1.46	0.393	13.791	1	0.000	0.232	0.107	0.502
Duration of surgery (< 1 h)	−2.005	0.469	18.282	1	0.000	0.135	0.054	0.338
Previous C- section (yes)	2.009	0.402	24.986	1	0.000	7.457	3.392	16.395
Elective C-section	0.594	0.378	2.466	1	0.116	1.812	0.863	3.804

^a^ Variable(s) entered on step 1: age, place of living, co-morbidity, antibiotic use, duration of surgery, previous C-section, elective C-section

Age less than 35 years, reduces the chance for SSI development compared to the patients in the age-group 35 years and over > 35 years (RR 0.425; 95% CI; 0.199–0.906 and *P* = 0.027). Patients with repeated C-section were 7.4 times more likely to develop SSIs compared to the patients with no history of previous C-section (RR 7.457; 95% CI; 3.392–16.3395 and P was 0.000). One or more co-morbidities were associated with 8 fold increased risk for SSI development compared to patients without co-morbidity (RR 8.428; 95% CI; 3.681–19.300 and *P* = 0.000). Preoperative use of antibiotics reduces the chance for SSI development compared to the patients without antibiotic administration (RR 0.232; 95% CI; 0.107–0.502 and *P* = 0.000).

Duration of the operation less than 1 h reduced the chance for SSI development compared to the patients in which the duration of surgery exceeded 1 h (RR 0.135; 95% CI; 0.054–0.338 and *P* = 0.000) (Table 2).

Furthermore, 77.5% of the patients were administered cefazolin and gentamycin postoperatively whereas 14.5% received a combination with cefazolin, gentamycin and metronidazole as per hospital protocol, postoperatively (data not shown).

Multivariable logistic regression analysis confirmed that one or more co-morbidity, previous C- section, preoperative antibiotics and duration of the surgery < 1 h were predictors of SSI (Table 3).

**Table 3:** Multivariable logistic regression analysis for risk factors associated with SSI at the UCC of Kosovo, Clinic for Obstetrics and Gynecology

Variables ^a^	B	S.E.	Wald	Df	Sig.	Exp(B)	95% C.I. for EXP(B)	
							Lower	Upper
Age < 35	0.815	0.469	3.019	1	0.082	2.26	0.901	5.667
Co-morbidity (yes)	1.995	0.5	15.929	1	0.000	7.354	2.761	19.593
Antibiotics (yes)	−1.509	0.454	11.03	1	0.001	0.221	0.091	0.539
Duration of surgery (< 1 h)	− 1.31	0.55	5.666	1	0.017	0.27	0.092	0.793
Previous C-section (yes)	1.296	0.508	6.513	1	0.011	3.654	1.351	9.887

Multivariable logistic regression analysis confirmed that one or more co-morbidity, previous C-section, preoperative antibiotics and duration of the surgery < 1 h are predictors of SSI

^a^ Variable (s) entered on step 1: age, co-morbidity, antibiotics, duration of surgery, previous C-section

The mean day, in which SSI was diagnosed, was 10.3 ± 5.7 postoperative day, minimum 4 days and maximum 25 days while the median time to SSI was 7th postoperative day.

The microbiological profile revealed *Staphylococcus aureus* as the most frequent isolated pathogen in 28.1% (*n* = 9), followed by the second most common agent *Enterococcus faecalis* in 15.6% (*n* = 5) and *Escherichia coli* isolated in 9.4% (*n* = 3). There were 2 cases or 6.25% of clinical infection with negative (sterile) culture and in 2 cases combined microbial infection was identified, namely one case with *Escherichia coli* and *Serratia marcescens* and another case with *Escherichia coli* and *Proteus mirabilis* (Table 4).

**Table 4:** Microbiological profile in patients diagnosed with SSI^a^

Wound swab- bacteriological profile	Count	Percent
*Coagulase negative staphylococci spp.(CoNS)*	2	6.25
*Staphylococcus aureus*	9	28.1
*Serratia marcescens*	1	3.1
*Enterococcus faecalis*	5	15.6
*Negative culture (sterile)*	2	6.25
*Klebsiella pneumoniae*	1	3.1
*Missing (not taken)*	4	12.5
*Bacillus spp.*	1	3.1
*Pseudomonas aeruginosa*	1	3.1
*Escherichia coli, Proteus mirabilis*	1	3.1
*Escherichia coli*	3	9.4
*Escherichia coli, serratia marcescens*	1	3.1
*Klebsiella spp.*	1	3.1
Total	32	100

^a^Surgical site infection (SSI)

## Discussion

This study evaluated the rate of SSI after C-sections, determined risk factors for SSI development and identified the common bacteriological profile in the study population. Reported rates for SSI after C-section varied from 3 to 15% [13] causing substantially high maternal morbidity and mortality as well as prolonged hospital stays while increasing financial costs.

Overall the SSI rate in our current study (9.85%) was noticeably high, but yet comparable with the reported SSI rate globally. There is a wide range globally of reported SSI after C-sections varying from a SSI rate of 2.7% in a retrospective study conducted in Nova Scotia [14] to 5.5% in the USA [15] followed by high incidence rate of SSI up to 48% in low-resource settings in a Tanzanian tertiary hospital [16] 23.5% in Brazil [17] 18.8% in Malaysia [18] and 14.4% in Jordan [19]. A study from Saudi Arabia reported a SSI rate of 9.5% after cesarean delivery [20] while in a cross sectional survey conducted in the Estonian University Hospital reported a SSI rate after C-section of 6.2% [21]. These studies demonstrate that the overall SSI rate differs widely, based on the study sample, preexisting diseases, use of antibiotics as well as reliable methods for SSI documentation and reporting. Data from this study showed that independent risk factors for SSI development after C-section include: co-morbidity, previous surgery, preoperative antibiotics and duration of the surgery. There are conflicting results regarding the relationship between age and increased risk for SSI [22]. In a study conducted by Kaye et al. age was identified as a strong predictor for SSI. A significant correlation was reported between the increased age and increased risk for SSI [23]. In the current study, age less than 35 years, reduced the chance for SSI development compared to the patients in the age-group 35 years and over > 35 years (RR 0.425; 95% CI; 0.199–0.906, *P* = 0.027).

Our findings are comparable to other studies in which increased age along with decreased host immunity and associated co-morbidities increases risk for SSI occurrence.

Our data confirmed that the patients with a history of previous cesarean section were 7.4 times more likely to develop SSI compared to the group without prior cesarean surgery. Additionally we found that duration of surgery less than 1 h had a protective effect for SSI prevention. Data from the present study were in accord with a study conducted by Killian et al. in which duration of operation > 1 h posed increased the risk for SSI development after C-Section and that prolonged surgery time is an independent risk factor for the development SSI [24]. Other studies reported similar results revealing a significant correlation between the duration of the surgical procedure and wound infection [25]. Furthermore, prolonged surgery, lasting more than 3 h, was associated with a 4 fold increased risk for SSI occurrence [26]. An independent relationship between prolonged operative procedures and medical and surgical complications as well as venous thromboembolism was consistently reported [27]. The major explanations for these correlations include anesthesia-related stress, extensive tissue trauma and inadequate serum and tissue concentration of the antibiotics in prolonged surgical procedures [26].

The majority of the infections in our study sample were superficial infections (93.75%) whereas 6.25% were deep primary incisional surgical site infections. Organ/Space SSI or endometritis based on patient-reported criteria (uterine tenderness, abdominal pain and purulent discharge from the uterus) as defined by Wloch et al. [28] were not identified in the study subjects.

Cases were treated by incision, drainage, and wound dressing as needed on a daily basis, antibiotics were administered based on the antibiogram and wounds were left to close by secondary intention. In one patient, Type I necrotizing fasciitis was promptly diagnosed and treated surgically. The median time to SSI for all infections was 7th postoperative day and the mean day in which SSI was diagnosed was 10.3 ± 5.7 postoperative day.

As previously reported, there is an increased risk for SSI in the presence of other comorbidities [29] explicitly: anemias, obesity [30] hypertension, diabetes mellitus as well as other associated morbidities in the patient [31].

There is strong evidence of the protective role of antibiotic prophylaxis to reduce the SSI rate with remarkable low SSI incidence rate among the patients with antibiotic administration prior to surgery [32]. The main source of error in the clinical setting is the choice of the antibiotics related to procedure as well as dosing of the antimicrobials [32]. The American Society of Health-System Pharmacists recommends intravenous use of 2 g Cefazolin for patients weighing less than 120 kg and 3 g for patients > 120 kg as a prophylactic measure to prevent SSI occurrence [33]. While the timing and the dosing was a bit controversial, there are convincing data that administration of antibiotic 30 min prior to incision compared to the administration 60 min before incision is not associated with significant difference regarding the SSI rate [34]. It is important to note that the internal hospital policy in our Clinic to provide antibiotic prophylaxis before C-section is not in accordance with the Society of Obstetricians and Gynecologists of Canada (SOGC) clinical practice guidelines for antibiotic prophylaxis in obstetric procedures [12] does not follow the recommendations of the American Society of Health-System Pharmacists [33] and is not in compliance with the Royal College of Obstetricians and Gynecologists (RCOG) clinical guidelines for caesarean section [35]. RCOG recommends intravenous use of 750 mg cefuroxime for women with no penicillin allergy. For women with penicillin allergy, intravenous dose of metronidazole 500 mg or gentamycin 160 mg are recommended [35].

Differences in our hospital policy regarding the antibiotic prophylaxis before C-section with other clinical guidelines, are mainly caused by the lack of adequate prenatal care in pregnant women, absent or insufficient screening for common bacterial infections in pregnancy and urinary tract infection, low socioeconomic status of the patients and importantly by our local list of essential medicines. There is non-compliance of our policy with RCOG guidelines in terms of duration of the antibiotic administration and broad spectrum regimen with gentamycin and metronidazole for cases of prolonged surgery, excessive blood loss and presence of associated risk factors. Additional doses of antibiotics are given postoperatively without Microbiology advise up to the third postoperative day. Therefore, adherence to the evidence-based practice guidelines and a review of our current hospital policy for antibiotic prophylaxis, presents a future challenge in our setting.

Our study demonstrated that in 65.5% of the operated patients, Cefazolin was used prophylactically and that preoperative use of antibiotics reduced the chance for SSI development compared to the patients without antibiotic administration (RR 0.2325; 95% CI; 0.107–0.502, *P* = 0.000). However, data from this study reveal serious weaknesses in the current internal hospital protocols regarding the preoperative prophylaxis with antibiotics as well as postoperative treatment. CDC and updated Prevention Guidelines 2017 strongly recommend that for clean and clean-contaminated procedures no supplementary prophylactic antimicrobial administration is needed if the skin incision is closed in the operating room even if there a drain is left [4].

Considering this issue, 77.5% of our patients received a combination of Cefazolin and gentamycin postoperatively for 3 days, whereas 14.5% received a combination with Cefazolin, gentamycin and metronidazole postoperatively as per hospital protocol. This was not supported by the evidence-based recommendation for the prevention of SSI (data not shown).

While all patients received antibiotics for more than 24 h after surgery, this practice showed no protective effect, therefore prolonged use of antibiotics did not reduce SSI incidence rate and can further add to microbial resistance in our setting.

Similar to the study conducted by Kaplan et al. which exposed *Staphylococcus aureus* in 42% of positive samples after cesarean delivery [36] the bacteriological profile in patients with SSI in our study sample, showed predominance of the skin microflora mostly *Staphylococcus aureus* (28.1%) and *Coagulase negative staphylococci spp. (CoNS)* to a lesser extent (6.25%) which is expected for the wound class I.

Similar to our results, in a prospective evaluation for wound infection in more than 2000 cases after C-section, authors reported *Enterococcus faecalis* (17%) as the second most prevalent pathogen [37]. These findings support our data as *Enterococcus faecalis* was isolated in 15.6% of the positive cultures in our study subjects.

In the current study, isolation of the gram negative pathogens commonly observed in wound class II, should increase our awareness of the potential sources of infection, exogenous contamination, medical staff carriers and environmental factors [32].

### Limitations of the study

A limitation of the current study is the small sample size due to the short study period. Maternal factors such as prenatal care, multiple gestation, labor disorders, the rate of gestational diabetes versus pre-existing diabetes and prevalence of urinary tract infections after C-section were not included in the present study, hence findings cannot be generalized. A larger cohort study would further address some of these issues.

The CDC-NNIS risk index System was not included in the analyses because we had no wound class II, III and IV as well as no ASA scores 3 and 4.

This study provides initial baseline data for the SSI rate, risk factors and microbial profile at the Clinic for Obstetrics and Gynecology, Kosovo.

## Conclusions

The high incidence rate of the SSIs in the current study highlights the need for prioritizing SSI control by creating methods for clear post discharge surveillance at a national level by developing patient and physician-based valid measures for SSI evaluation after discharge. Programs for wound surveillance can decrease the rate of infection which in turn may decrease costs of surveillance and treatment.

Identifying and properly managing patients with comorbidities and reducing surgery time can further decrease SSI rates. Introducing a new and evidence-based policy regarding the preoperative and postoperative antibiotics remains a cornerstone for the microbial resistance prevention while providing optimal protective effect for the patients, and hence reducing the high costs of prolonged antibiotherapy in our country. The National Committee for the Prevention and Control of Nosocomial infection in Kosovo should provide updated guidelines for the control and prevention of the nosocomial infections based on systematic surveillance.

## Data Availability

The datasets generated and analyzed during the current study are not publicly available because they are archived in the database of the University Clinical Center of Kosovo, Obstetrics and Gynecology Clinic and only are used for scientific purposes. Datasets may be available from the corresponding authors upon request.

## Abbreviations

ASA: American Society of Anesthesiologists
CDC: Center for Disease Control and Prevention
CS: Cesarean section
HAIs: Health-care associated infections
NHSN: National Healthcare Safety Network
SOGC: Society of Obstetricians and Gynecologists of Canada.
SPSS: Statistical Package for Social Sciences
SSI: Surgical site infections
UCCK: University Clinical Center of Kosovo
WHO: World Health Organization
